# Exoscope for Upper Extremity Peripheral Nerve Surgery: Revision Carpal Tunnel Release With Epineurolysis and Hypothenar Fat Flap

**DOI:** 10.7759/cureus.22539

**Published:** 2022-02-23

**Authors:** Nelson A Rodriguez-Unda, Daniel S Wu

**Affiliations:** 1 Plastic and Reconstructive Surgery, Baylor Scott & White Medical Center-Temple, Temple, USA; 2 Plastic Surgery, Central Texas Veterans Health Care System, Temple, USA

**Keywords:** exoscope, hypothenar fat flap, peripheral nerve surgery, epineurolysis, carpal tunnel syndrome, microsurgery

## Abstract

The exoscope is a high-resolution three-dimensional external microscope that allows the surgeon to perform delicate dissection in multiple regions of the body. The exoscope was originally used for intracranial and spine surgery. In this article, we describe its novel use in upper extremity peripheral nerve decompression surgery after recurrent carpal tunnel syndrome. This surgery is typically performed under the microscope, which allows precise microsurgical dissection to distinguish scar tissue from healthy nerve fascicles. Our case report highlights a 70-year-old man with recurrent carpal tunnel syndrome who underwent revision carpal tunnel surgery with epineurolysis and hypothenar fat flap. The ergonomic benefits of using the exoscope for microsurgery are described, along with intraoperative photos. Adequate symptom resolution was achieved.

## Introduction

The exoscope (extracorporeal operating microscope) system is a video camera mounted onto a stereotactic arm that is positioned outside the body and then projected onto the surgical field. The image is projected for the surgeon onto a video/computer monitor in real time [1,2]. The ORBEYE (Olympus, Tokyo, Japan) is the first exoscope with 4K high-definition clarity with concurrent three-dimensional (3D) visualization [2,3]. The optical zoom ranges from 1.1 to 25.8x magnification, the focal lengths range from 220 to 550 mm, and the fields of view range from 7.5 to 171 mm [3]. The exoscope was initially used in neurosurgery for intracranial surgery [4-8], spinal surgery [3,5,8], carotid endarterectomy [5,8,9], and tarsal tunnel release [5]. Its role has expanded to include otolaryngology [10], general surgery [11,12], and plastic surgery [13]. In the latter, the exoscope has been used for flap dissection and microsurgical anastomoses [13]. However, only three cases of peripheral nerve surgery using the exoscope have been described to date, all of which occurred in the lower extremity [5]. We present the first upper extremity nerve decompression surgery using the exoscope.

## Case presentation

The patient was a 70-year-old, right-hand-dominant male with the following comorbid conditions: coronary artery disease, active tobacco use, history of pulmonary embolism on chronic anticoagulation, hypertension, and hyperlipidemia. He presented to the clinic with a 14-month history of left-sided hand pain, localized in the wrist and fingertips of the left thumb, index, and long finger. He described throbbing pain rated as 4 out of 10 in severity that woke him up at night. Shaking and massaging his wrist was required for the pain to ameliorate. Conservative measures, consisting of a wrist splint and corticosteroid injection, had failed. The patient had previously undergone open carpal tunnel release 12 years before. He was interested in having a more definitive surgical treatment.

The patient displayed no acute distress and was alert and oriented. He had normal range of motion of the shoulders and elbows bilaterally. The left-hand fingers and thumb had a normal cascade. Heberden’s nodes were present in the index and long fingers. The thenar and hypothenar muscles were normal in tone and strength without atrophy. He had a positive Tinel’s sign in the left wrist and a positive Durkan’s sign; Phalen's test was positive as well; and two-point discrimination was abnormal with more than 8 mm in the index, long, and thumb fingertips. He reported no pain at the first carpometacarpal joint on the grind test.

Electromyography and nerve conduction velocity (EMG-NCV) testing demonstrated severe median nerve compression at the level of the wrist. There was no sensory signal recorded at the left index finger. The patient had decreased motor neuron recruitment of the abductor pollicis brevis.

Surgical procedure

Standard open carpal tunnel markings were performed. We proceeded with sharp dissection of the skin and subcutaneous tissue, taking care to avoid any injury to the median nerve. The median nerve was proximally and distally identified in the unscarred tissue and then freed from the roof of the carpal tunnel. The operative plan consisted of left revision open carpal tunnel in addition to microsurgical epineurolysis and hypothenar fat flap.

**Figure 1:**
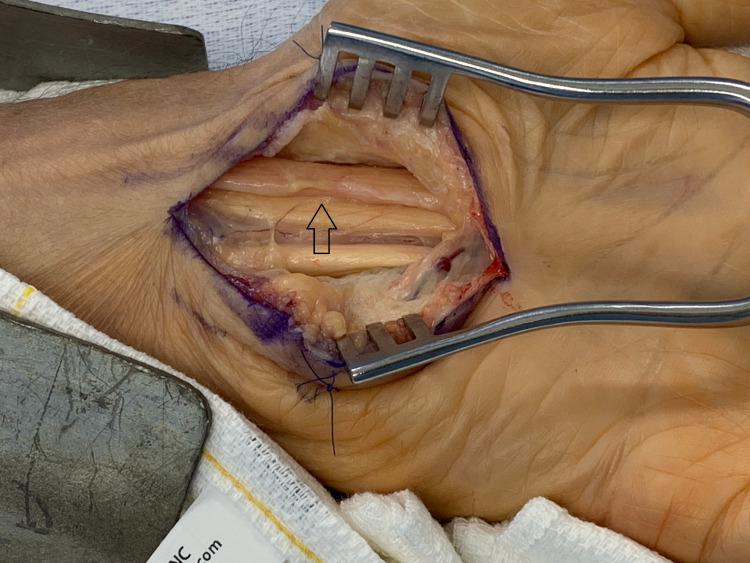
Recurrent carpal tunnel syndrome with hourglass scar tissue at the level of the wrist The arrow points at the site of the distal forearm fascia with an underlying hourglass-shaped compression site.

We identified an hourglass constriction deformity of the median nerve with associated nerve contusion at the level of the distal forearm fascia (Figure 1). Epineurolysis was subsequently performed using the 4K-3D exoscope. We performed longitudinal epineurolysis along the length of the median nerve from the distal forearm to the palmar arch, followed by transverse epineurotomy to fully release the nerve. The endpoint was visualization of the normal nerve fascicles with no further constriction of the median nerve (Video 1).

**Video 1:** 4K three-dimensional (3D) epineurolysis of the median nerve performed with the exoscope Microsurgical instruments under high magnification are used to release the nerve fascicles from the scar.

**Figure 2:**
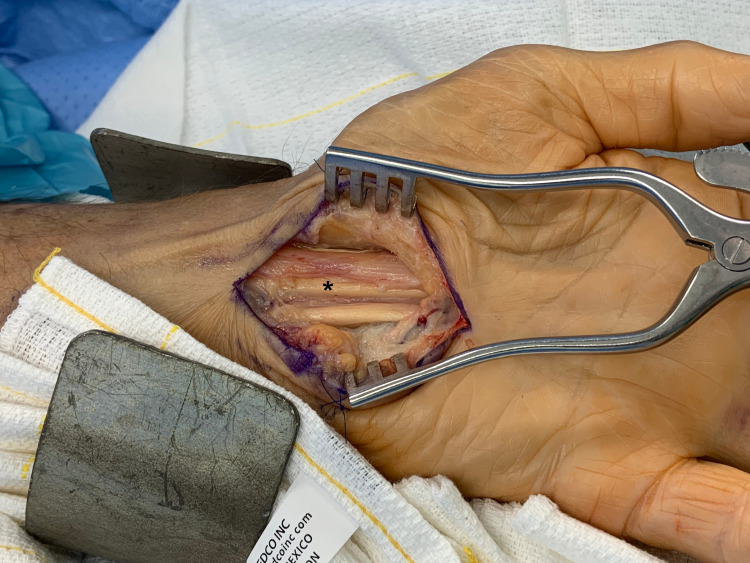
Nerve is decompressed until normal fascicles are seen and scar tissue has been removed The asterisk indicates contused median nerve (after chronic compression) after the longitudinal and transverse epineurolysis has been completed.

As seen in Figure 2, the goal of the dissection after transverse and longitudinal epineurolysis is the identification of normal appearing neurovascular bundles. Due to the chronicity of the compression, we can observe segmental median nerve contusion. At the end of the procedure, a hypothenar fat flap with small perforators based on the ulnar artery was used to provide a well-vascularized surface between the nerve and skin [14]. Finally, the incision was closed using 4-0 Prolene. There were no complications.

## Discussion

We present a novel description of the use of a high-resolution 4K-3D exoscope in upper extremity nerve decompression in the setting of revision surgery. The exoscope provided excellent visualization of the surgical field, as we were able to delineate the normal fascicles of the median nerve from the scar tissue and healthy epineurium. In addition, we were able to perform epineurolysis with 3D depth of vision with high magnification. The exoscope offered a wider field of vision, so the instruments could be used and passed easily. From the surgeon’s standpoint, the exoscope is more ergonomic because the cervical and lumbar spines are in the neutral position while looking straight ahead to perform the surgery. Since they can see the surgery unfold on the screen, the surgical team, surgical scrub technician, and circulating nurse were more engaged and responded to the surgeon’s needs proactively rather than reactively with each step of the procedure. In this case, the patient reported complete resolution of his nocturnal pain with improving hand sensation as measured by improved two-point discrimination 5 mm in the index and long fingers.

**Figure 3:**
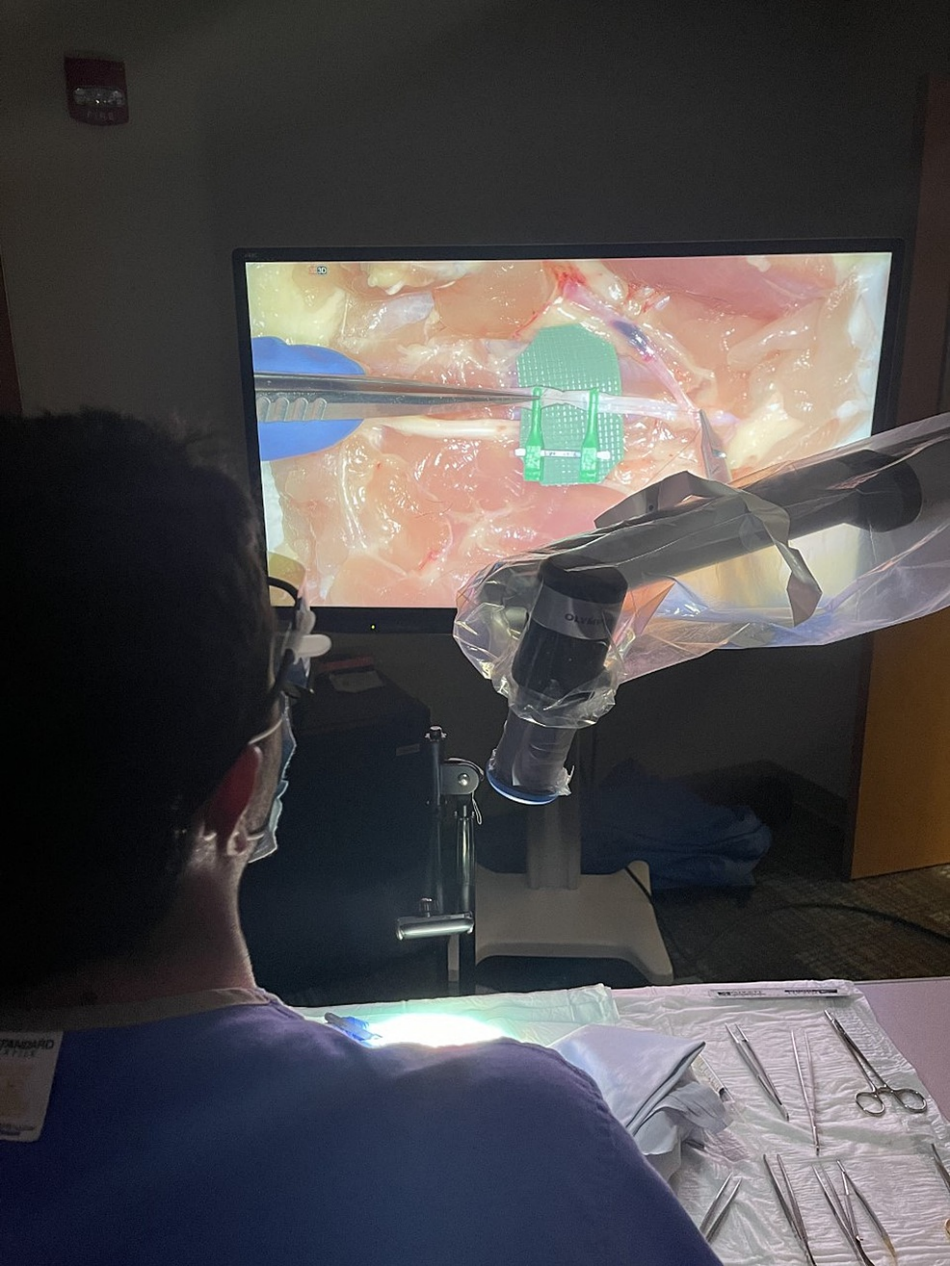
Exoscope arm setup during microsurgical anastomosis Note the neck in neutral position and the wide field of view in front of the surgeon, there is ample space in the operating field to do surgical exposures and pass instruments. On the right, we can observe the exoscope arm approximately 40 cm away from the anastomosis.

The benefits of the exoscope have been described and include improved comfort and ergonomics of the surgeon and first assistant, wider field of vision and large focal distance/depth of field, excellent image quality and magnification (Figure 3), improved engagement of the operating room staff, and small footprint of the ORBEYE [3-5,11,12,14,15]. Because of the shared image of the assistant and surgeon, there is improved communication, hands-on participation, and teamwork. One drawback to this method is the reversal of the assistant’s movements because the assistant and the surgeon are facing each other and using the same monitor. This can be overcome with a proper operating room setup that allows for the assistant and surgeon to be on the same side or for the use of a secondary monitor [12,14].

## Conclusions

We presented the first description of nerve decompression surgery in the upper extremity using the 4K-3D exoscope. The 4K-3D provided clear, excellent visualization to delineate the anatomic details of the normal fascicles of the median nerve, epineurium, and scar tissue. The exoscope (ORBEYE) could be used as an alternative to the traditional surgical microscope in nerve decompression surgeries of the upper extremity, especially in cases in which fine microsurgical dissection is needed.
